# CT-based radiomic nomogram for predicting the severity of patients with COVID-19

**DOI:** 10.1186/s40001-022-00634-x

**Published:** 2022-01-25

**Authors:** Hengfeng Shi, Zhihua Xu, Guohua Cheng, Hongli Ji, Linyang He, Juan Zhu, Hanjin Hu, Zongyu Xie, Weiqun Ao, Jian Wang

**Affiliations:** 1 Department of Radiology, Anqing Municipal Hospital, Anqing, Anhui China; 2 Department of Radiology, Tongde Hospital of Zhejiang Province, No. 234, Gucui Road, Hangzhou, Zhejiang China; 3 Jianpei Technology, Hangzhou, Zhejiang China; 4 Department of Radiology, The First Affiliated Hospital of Bengbu Medical College, Bengbu, Anhui China

**Keywords:** COVID-19, X-ray computed tomography, SARS-CoV-2, Radiomics

## Abstract

**Background:**

The coronavirus disease 2019 (COVID-19) is a pandemic now, and the severity of COVID-19 determines the management, treatment, and even prognosis. We aim to develop and validate a radiomics nomogram for identifying patients with severe COVID-19.

**Methods:**

There were 156 and 104 patients with COVID-19 enrolled in primary and validation cohorts, respectively. Radiomics features were extracted from chest CT images. Least absolute shrinkage and selection operator (LASSO) method was used for feature selection and radiomics signature building. Multivariable logistic regression analysis was used to develop a predictive model, and the radiomics signature, abnormal WBC counts, and comorbidity were incorporated and presented as a radiomics nomogram. The performance of the nomogram was assessed through its calibration, discrimination, and clinical usefulness.

**Results:**

The radiomics signature consisting of four selected features was significantly associated with clinical condition of patients with COVID-19 in the primary and validation cohorts (*P* < 0.001). The radiomics nomogram including radiomics signature, comorbidity and abnormal WBC counts showed good discrimination of severe COVID-19, with an AUC of 0.972, and good calibration in the primary cohort. Application of the nomogram in the validation cohort still gave good discrimination with an AUC of 0.978 and good calibration. Decision curve analysis demonstrated that the radiomics nomogram was clinically useful to identify the severe COVID-19.

**Conclusion:**

We present an easy-to-use radiomics nomogram to identify the patients with severe COVID-19 for better guiding a prompt management and treatment.

## Background

The coronavirus disease 2019 (COVID-19), which is caused by severe acute respiratory syndrome coronavirus 2 (SARS-CoV-2), has widely spread all over the world [1–3] due to person to person transmission and it could be described as a pandemic [4]. SARS-CoV-2 epidemic has attracted worldwide attention and caused a certain degree of social panic.

The incidence and mortality of COVID-19 varied in different countries or territories [1]. SARS-CoV-2 has major symptoms (fever, dyspnea, cough) and minor symptoms (fatigue, dysgeusia, anosmia, gastrointestinal symptoms, headache and skin lesions) [5–7]. According to the previous literature [8, 9], 81% of the patients with COVID-19 had mild symptoms, but it was the rest of the patients (19%) who were in severe and critical conditions that determined the mortality. Because the severe patients with COVID-19 directly influence the clinical management and treatment [10], it is crucial for clinicians to evaluate the condition of COVID-19.

Chest computed tomography (CT) can serve as an important modality to screen, diagnose and evaluate COVID-19 [11, 12]. It was reported that the CT features of COVID-19 was manifested as patchy ground-glass opacities (GGOs) with or without consolidation distributed in subpleural areas of bilateral lungs [11], and increased numbers, greater extent of consolidation on chest CT images were related to progression of COVID-19 [12]. However, these studies were limited to qualitative analysis, merely focusing on the manifestation of COVID-19 on chest CT images to screen potential new cases of COVID-19. Some scholars [13, 14] applied quantitative scores of imaging examinations to assess the severity of COVID-19 with active results. Baratella et al. [13] used a semi-quantitative score of chest X-ray to assess the severity of lung involvement in COVID-19 patients. Score 0–4 represented different severities of lung involvement, respectively; results showed that the severity score based on chest X-ray can predict the clinical progression in cases that scored 0, 3, or 4. Trias–Sabrià et al. [14] used lung ultrasound score to assess the severity of COVID-19; they found that lung ultrasound score ≥ 24 points can help identify the severe COVID-19 patients. The quantitative analysis of correlation between pulmonary abnormalities of COVID-19 on chest CT images and the clinical severity or condition of COVID-19 has not been investigated thoroughly, which may be promising for improving the management of COVID-19.

As the wide application of the artificial intelligence technology for the detection of pulmonary nodules has demonstrated great success [15], computer-aided detection and analysis makes quantification and classification of COVID-19 possible. The artificial intelligence evaluation system of COVID-19 was rapidly developed and applied to solve the insufficient expertise of radiologists and speed up screening potential new cases of COVID-19 [15].

Radiomics, as an emerging technique involved with the extraction of high-throughput data from quantitative imaging features and the subsequent association of these parameters with clinical data, has been applied in various diseases. For instance, CT-based radiomics has shown a good performance in predicting of recurrence, metastasis and treatment effect of tumors [16, 17]. As far as we know, the existing literature mainly focuses on discriminating COVID-19 and non-COVID-19 pneumonia or other types of viral pneumonia, the predicted results were comparatively ideal [18, 19]. The average overall case-fatality rate was 2.3% in confirmed COVID-19 patients, but that was up to 49.0% in severe patients [20]. Little literature has reported the application of radiomics for evaluation of the severity of COVID-19. If CT-based radiomics can identified the severity of COVID-19, it may guide the clinical treatment and benefit patients. Therefore, the purpose of this study was to apply the artificial intelligence to quantitatively analyze the lung abnormalities associated with COVID-19, and to develop and validate a radiomics nomogram for identifying the severity of infection in COVID-19 patients who need better management in intensive care units.

## Methods

### Patients

The protocol for this study was approved by the Institutional Review Board of Anqing Municipal Hospital. All patients or their legally authorized representatives provided written informed consent prior to participation in this study. A total of 260 patients with COVID-19 from three hospitals (Anhui and Zhejiang, China) were enrolled from 24 January 2020 to 1 March 2020. All patients were grouped into primary cohort and validation cohort using stratified random resampling method with a ratio of 3:2. Primary cohort data were used to establish the prediction model, which was then validated by the validation group data. The inclusion criteria were (1) positive for RT-PCR test of SARS-CoV-2; (2) complete clinical data; (3) patients underwent a CT scan. The exclusion criteria were (1) low-quality images and (2) normal CT. Their baseline clinical and image data were reviewed retrospectively.

### Clinical information

Basic information including gender, age, comorbidity (hypertension, diabetes mellitus, cardio-cerebrovascular disease, the history of surgery for important organs, etc.) and laboratory examinations including C reactive protein (CRP), white blood cell (WBC) and lymphocytes were derived from medical records for all patients. A score system based on the number of comorbidities was used to evaluate the state of the patients: none has a score of 0, one has a score of 1, two has a score of 2, and more than two has a score of 3.

According to the guideline of American Thoracic Society Criteria [10], the clinical condition of the patients was classified into non-severe (mild and common) and severe (severe and critical) types. Severe patient was defined as (1) respiratory frequency ≥ 30 breaths/min; (2) finger oxygen saturation in resting state ≤ 93%; (3) respiratory distress; (4) the presence of shock; (5) arterial oxygen tension (PaO_2_) ≤ 300 mmHg; (6) respiratory failure requires mechanical ventilation; (7) patients with organ failure need ICU monitoring and treatment.

### CT image acquisition, segmentation, and quantitative analysis

All patients underwent chest CT scan using a multidetector scanner (16-MDCT, SOMATOM Emotion16, SIEMENS, Germany; 16-MDCT, Definition AS, SIEMENS, Germany; 64-MDCT, Optima CT680, GE, USA; 16-MDCT, Light Speed, GE, USA) with the following parameters: display field of view (dFOV) 32 cm, 300 mAs and 120 kV, and slice thickness 5 mm. All CT images were acquired at deep inspiration in the supine position and reconstructed with the slice thickness of 0.625–1.25 mm.

Pleural thickening and pleural effusion were observed in mediastinal window with a window width of 350 Hounsfield unit (HU) and a window level of 40 HU.

**Fig. 1:**
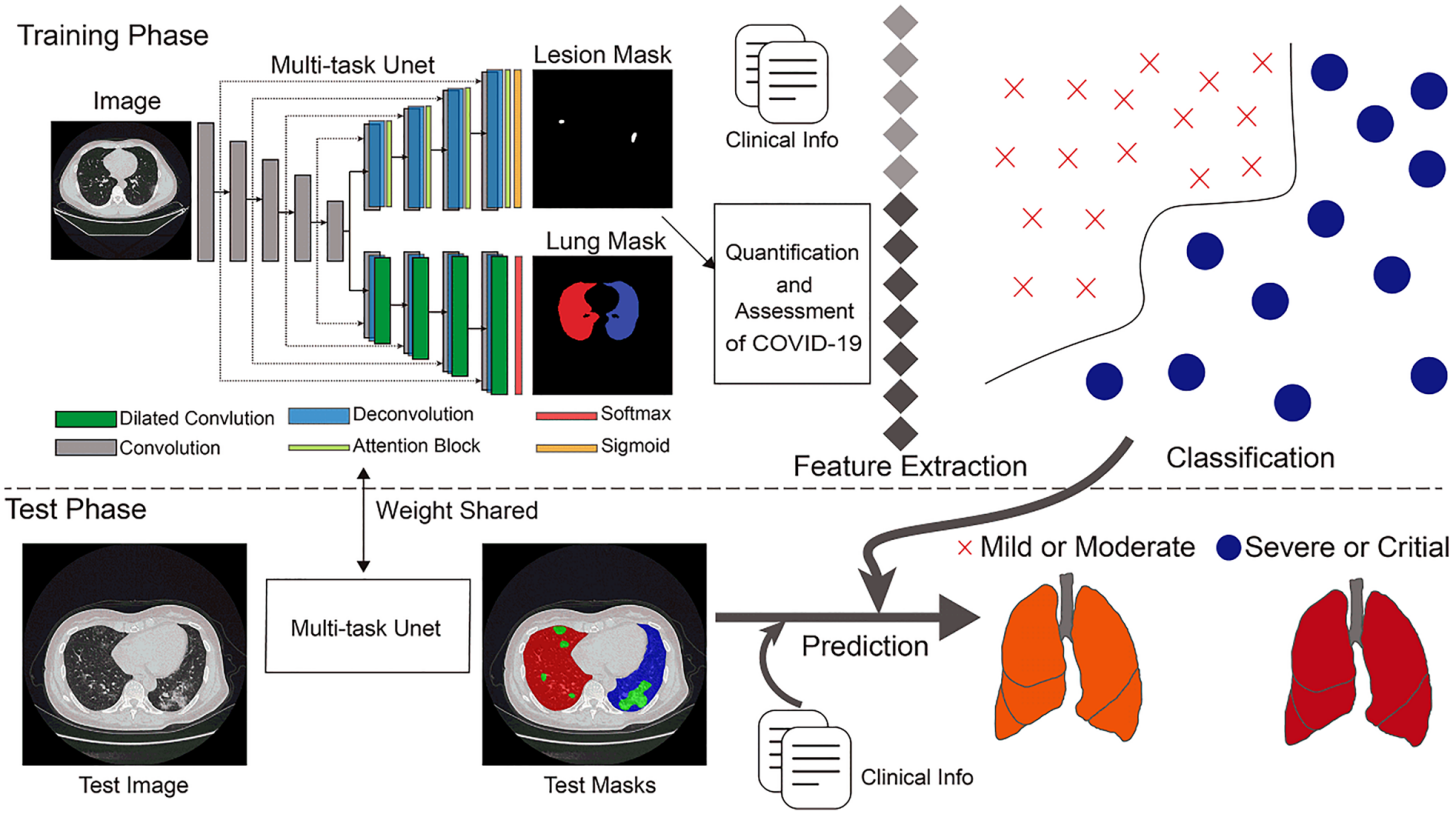
Flowchart of radiomics procedure in this study

The flowchart of radiomics procedure is shown in Fig. 1. All CT images of COVID-19 were segmented by a pre-trained multi-task Unet network. Multi-task Unet is a 2D Unet [21]-based network with a single encoder and two parallel decoders, one decoder with attention block to learn the lesion segmentation and another decoder with stacked dilated convolutions to learn the lung segmentation, providing at the same time a more efficient feature encoding and a regularizing effect. At each decoder layer, the features from the corresponding encoder layer are concatenated which helps in retaining multi-scale features. Specifically, we concatenated encoder and decoder features, based on the attention block (integration of spatial attention and channel attention) [22] which was learnt for encoder feature and decoder feature separately. By facilitating joint primary of two tasks, not only the model size and inference time were greatly reduced, but also low-level features were effectively reused. The network primary and inference of this experiment were implemented based on Dr. Pecker cloud platform (http://www.jianpeicn.com/category/yuepianjiqiren). It is available to public research institutions and is free now around the world for COVID-19 research analysis and prevention.

**Fig. 2:**
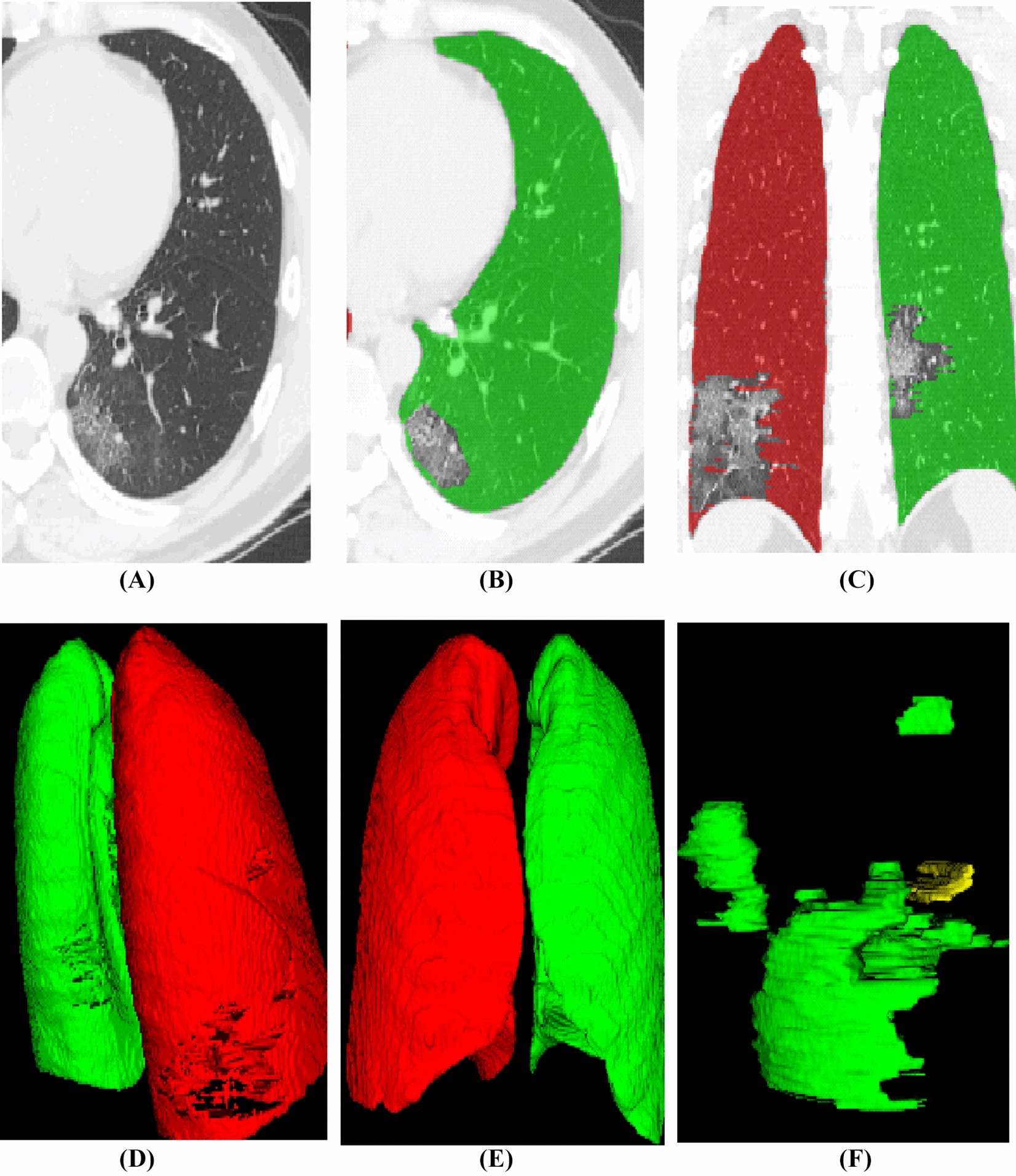
Illustration of imaging segmentation. **A** Axial view of CT image; **B**, **C** delineation of lung abnormalities; **D**, **E** corrosion map, **D** indicates an involvement of dorsal lung, **E** shows non-involvement in the anterior part of lung; **F** 3D regions of interests

Primary samples with detailed delineation of each lesion and lung regions were required. Two cardiothoracic radiologists who had 5–15 years’ experience segmented the lesion and lung region using ITK-SNAP software (version 3.8.0; http://www.itksnap.org) in lung window with a width of 1500 HU and a level of 600 HU. The margin of the lesion was delineated for each axial slice (Fig. 2A–C). Then a 3D region of interests (ROI) was obtained (Fig. 2D–F). We split 650 annotated CT scans into 550 for primary and 100 for testing. We tested our model on a holdout 100 CT scans as well to illustrate the robustness of our proposed approach. The average Dice similarity coefficient was 0.973 for the right lung, 0.985 for left lung, and 0.864 for lesion segments.

After segmentation by multi-task Unet, all segmentation results were manually reviewed again in this experiment. Various metrics were computed to quantify the COVID-19 lesion, including volumes of lesion in the whole lung, and volumes of lesion in each lung segment. The GGO and consolidation were distinguished with a threshold value of 450 HU. We used the SimpleITK software tools (http://www.simpleitk.org) to quantify the mean HU of lung and lesion, number and volume of lesions, volume of GGO and consolidation in double lungs, and the volume of the whole lung automatically. Simultaneously, the ratio of volume of GGO and consolidation in bilateral lungs to total lung volume and to lesion volume in bilateral lungs were calculated, respectively. Totally, there were 14 quantitative parameters acquired for feature selection and radiomics model construction.

### Statistical analysis

R software (version 3.0.1; http://www.Rproject.org) was used for statistical analysis. All the radiological features were normalized between 0 and 1. The ‘caret’ package was used to obtain the accuracy, sensitivity and specificity of model. ‘pROC’ package, ‘rms’ package and ‘rmda’ package were used to perform receiver operating characteristic (ROC) analysis, calibration curve analysis and decision curve analysis, respectively. Two-sided *P* < 0.05 indicated statistical significance.

### Radiological feature selection and radiomics signature construction

The most useful predictive features were selected using the least absolute shrinkage and selection operator (LASSO) method [23]. Briefly, the optimized hyperparameter *λ* was first determined using tenfold cross-validation with binomial deviance as a criterion. Then the features with non-zero coefficient were selected based on the determined optimal *λ*. Finally, LASSO regression was conducted to construct the radiomics signature and a radiomics score (Rad-score) was calculated for each patient via a linear combination of selected and weighted features by their corresponding coefficients.

### Individualized prediction model construction

Besides the radiomics features, the clinical data (termed “clinical feature” later in this article) were also collected. Two clinical features including comorbidity and abnormal WBC counts, which were significantly different between severe and non-severe COVID-19 in univariate regression analysis, were combined with Rad-score to build the nomogram using multivariate logistic regression. The nomogram provides the clinicians with a quantitative tool to predict individual probability of severe or non-severe COVID-19.

### Performance validation of the nomogram in the primary cohort

In the validation cohort, the same logistic regression formula formed in the primary cohort was used to calculate total points for each patient. Total points were then used as a factor for logistic regression analysis in validation cohort. Finally, two methods including calibration curve analysis and ROC analysis were used to evaluate the performance of nomogram model. Calibration curves were plotted to assess the agreement between the predicted event probability and the observed event probability. The ROC analysis was performed to evaluate the performance of the nomogram. Accuracy, sensitivity and specificity were calculated in both primary cohort and validation cohort.

### Clinical use

Decision curve analysis was performed to determine the clinical practicability of the nomogram by quantifying the net benefits at different threshold probabilities in both the primary and validation cohorts.

## Results

### Clinical characteristics

There were 156 and 104 patients with COVID-19 enrolled in primary and validation cohorts, respectively. Characteristics of patients in the primary and validation cohorts are shown in Table 1. There were significant differences in comorbidity, presence of pleural thickening, CRP increase, abnormal WBC and lymphocytes counts between severe and non-severe patients with COVID-19 in both primary and validation cohorts. Age and presence of pleural effusion differed between severe and non-severe patients with COVID-19 in primary cohort, while they did not differ in validation cohort. A significantly higher proportion of male with severe condition was shown in validation cohort; however, it did not show a significant difference in primary cohort. Note that nine severe patients presented with increased WBC counts.

**Table 1:** Characteristics of patients in the primary and validation cohorts

Characteristics	Primary cohort		*P* value	Validation cohort		*P* value
	Non-severe group (*n* = 130)	Severe group (*n *= 26)		Non-severe group (*n* = 86)	Severe group (*n* = 18)	
Male	68 (52.3)	18 (69.2)	0.171	40 (46.5)	14 (77.8)	0.031
Age	42.90 (14.71)	58.23 (16.23)	< 0.001	44.58 (14.37)	49.50 (13.10)	0.183
Comorbidity			< 0.001			0.004
0	106 (81.5)	11 (42.3)		67 (77.9)	11 (61.1)	
1	21 (16.2)	6 (23.1)		12 (14.0)	5 (27.8)	
2	3 (2.3)	5 (19.2)		7 (8.1)	0 (0.0)	
3	0 (0.0)	4 (15.4)		0 (0.0)	2 (11.1)	
Pleural thickening			< 0.001			< 0.001
No	85 (65.4)	4 (15.4)		57 (66.3)	1 (5.6)	
Yes	45 (34.6)	22 (84.6)		29 (33.7)	17 (94.4)	
Pleural effusion			0.001			0.12
No	124 (95.4)	19 (73.1)		80 (93.0)	14 (77.8)	
Yes	6 (4.6)	7 (26.9)		6 (7.0)	4 (22.2)	
CRP increase			0.004			0.002
No	63 (48.5)	4 (15.4)		41 (47.7)	1 (5.6)	
Yes	67 (51.5)	22 (84.6)		45 (52.3)	17 (94.4)	
WBC abnormal			< 0.001			< 0.001
No	106 (81.5)	12 (46.2)		73 (84.9)	7 (38.9)	
Yes	24 (18.5)	14 (53.8)		13 (15.1)	11 (61.1)	
Lymphocytes abnormal			< 0.001			0.006
No	76 (58.5)	3 (11.5)		48 (55.8)	3 (16.7)	
Yes	54 (41.5)	23 (88.5)		38 (44.2)	15 (83.3)	
Rad-score	0.095 (0.018, 0.222)	0.195 (0.083, 0.337)	< 0.001	0.051 (0.012, 0.134)	0.358 (0.207, 0.653)	< 0.001

### Feature selection and radiomics signature building

**Fig. 3:**
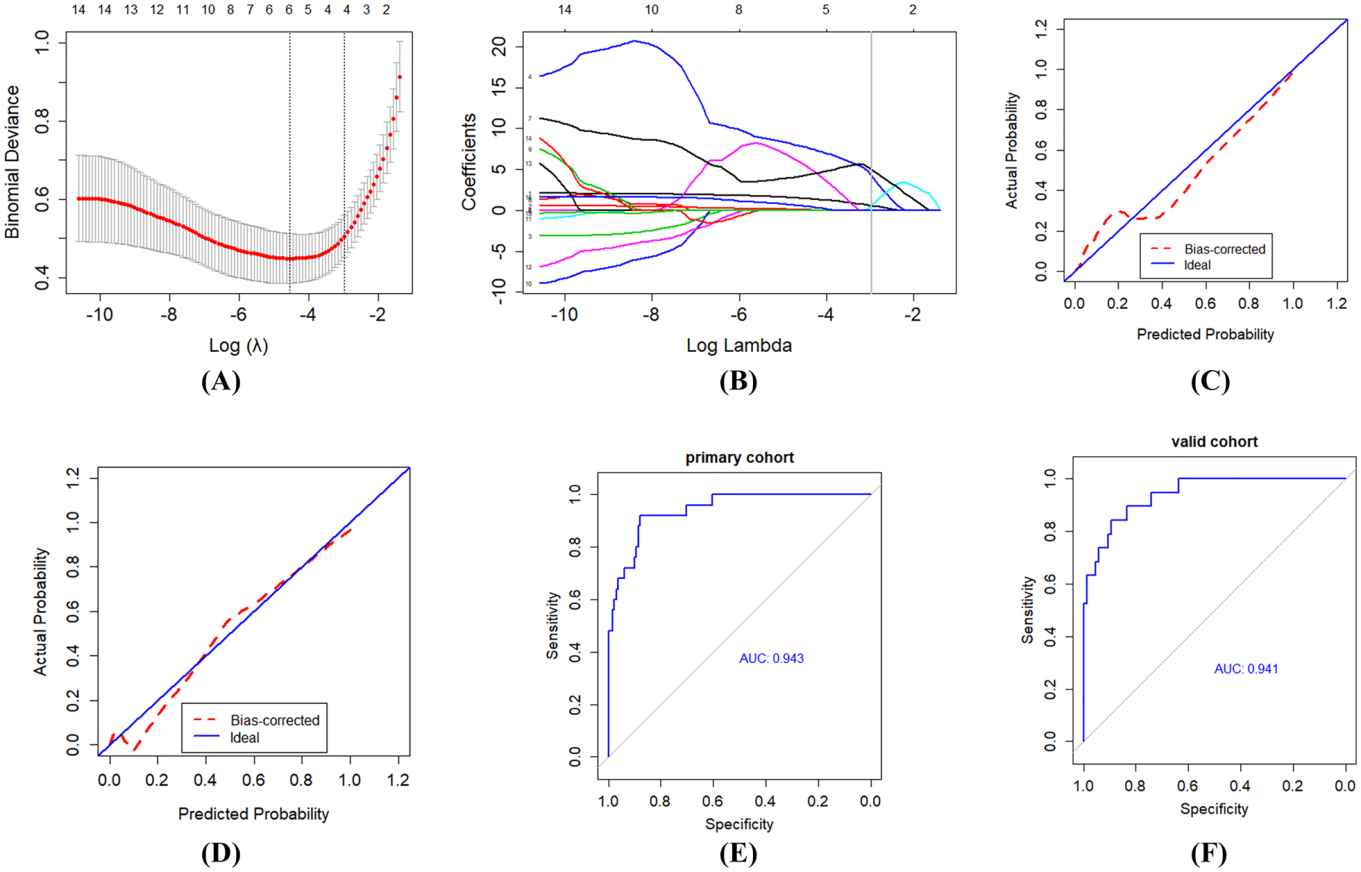
Feature selection and radiomics signature building using the least absolute shrinkage and selection operator (LASSO) binary logistic regression. **A** The parameter (*λ*) was screened using tenfold cross-validation method and parameter (*λ*) between two dotted lines was the optimal values using the minimum criteria and the 1 standard error of the minimum criteria (the 1SE criteria). **B **LASSO coefficient profiles of the 14 radiological features. A coefficient profile plot was conducted against the log (*λ*) sequence. *λ* value of 0.052, with log(*λ*) = − 2.96 was selected (1 − SE criteria) based on tenfold cross-validation. Vertical line was drawn at the value selected, which resulted in 4 non-zero coefficients. **C**, **D** Significant difference in radiomics score is shown between non-severe and severe groups in primary (**C**) and validation cohorts (**D**). **E**, **F** The receiver operating characteristic (ROC) curve for the radiomics signature. The calibration curves represent calibration of radiomics signature in terms of consistency between the predicted severe probabilities of COVID-19 and observed severe probabilities of COVID-19. The *x*-axis represents the predicted severe probabilities while the *y*-axis represents the actual probabilities. The actual probability was calculated by the formula PA = [1 + exp − (*ax* + *b*)]  1, where *x* = logit (*p*), *p* is the predicted probability, *a* is the slope estimates, and *b* is the corrected intercept. The 45° dotted line represents the perfect prediction of an ideal model and the dotted lines represents the performance of the built nomogram model, a closer fit to the dotted line represents a better prediction

After analysis of LASSO (Fig. 3A, B), four factors were selected from quantitative parameters: pleural thickening, total volume of the lesion, ratio of consolidation volume to whole lung volume and ratio of lesion volume to whole lung volume. The selected radiomics features and regression corresponding coefficients were recorded and then a radiomics score (Rad-score) was calculated and radiomics signature was built (Table 2).

**Table 2:** Four selected radiomic features and relevant coefficients in radiomics model

Radiomic features	Regression coefficient
Pleural thickening	0.044408621
Total volume of the lesion	0.424464103
Ratio of consolidation volume to whole lung volume	0.419327051
Ratio of lesion volume to whole lung volume	0.006363664

The radiomics signature was set up as Rad-score = 0.044408621 × pleural thickening + 0.424464103 × total volume of the lesion + 0.419327051 × ratio of consolidation volume to whole lung volume + 0.575642290 × ratio of lesion volume to whole lung volume + 0.006363664

### Diagnostic validation of radiomics signature

The model score was significantly different between non-severe and severe patients in the primary cohort (*P* < 0.001), which was further confirmed in the validation cohort (*P* < 0.001). The area under the ROC curve (Fig. 3C, D) for identifying the severe and critical patients based on the model was 0.943 and 0.941 in the primary and validation cohorts, respectively. In primary cohort, the accuracy, sensitivity and specificity for evaluation of the clinical condition were 0.885, 0.880 and 0.885, respectively. Correspondingly, the accuracy, sensitivity and specificity were 0.856, 0.842 and 0.859 in validation cohort. The calibration curve of the radiomics signature for the probability of severe and critical condition of COVID-19 patients indicated good agreement between prediction and observation in the primary cohort (Fig. 3E), which was then confirmed in the validation cohorts (Fig. 3F).

### Development of an individualized prediction model

**Fig. 4:**
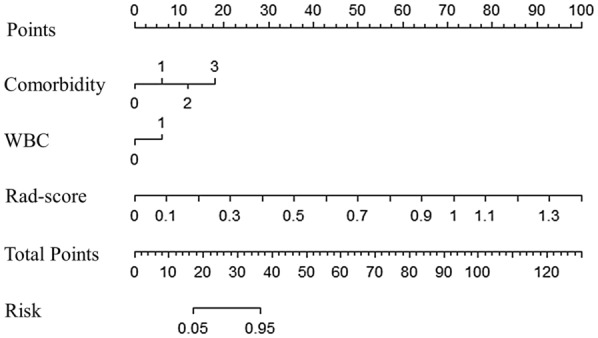
Nomogram for estimating the probability of severe type COVID-19. The radiomics nomogram was drawn with two clinical features (comorbidity and WBC: abnormal WBC counts) and radiomics score in the primary cohort. The point of each variable can be found based on the points’ uppermost scale, and the total points are the sum of points of each variable. The total points correspond to the bottom scale indicate the probability of severe COVID-19. For example, when total points are less than 17, the risk probability of severe COVID-19 is lower than 5%, and if the total points are higher than 37, the risk probability is greater than 95%

After a logistic regression analysis, the radiomics signature, comorbidity, and abnormal WBC counts were identified as independent predictors of the severity in COVID-19 patients (radiomics signature: OR 95% C.I. 2.137–5.742; comorbidity: OR 95% C.I. 1.192–2.087; WBC abnormal: OR 95% C.I. 8.472–311.790) (Table 3). The model combing the above three independent predictors were developed and presented as the nomogram (Fig. 4).

**Table 3:** Risk factors for clinical condition of patients with COVID-19

	Coefficient	*P* value	OR value	OR value (95% C.I.)	
				Lowest	Highest
Comorbidity	1.822	0.0014	1.577	1.192	2.087
WBC abnormal	1.882	0.0399	51.394	8.472	311.790
Rad-score	21.583	< 0.0001	3.940	2.137	5.742
Intercept	8.106	< 0.0001

### Validation of the radiomics nomogram

**Fig. 5:**
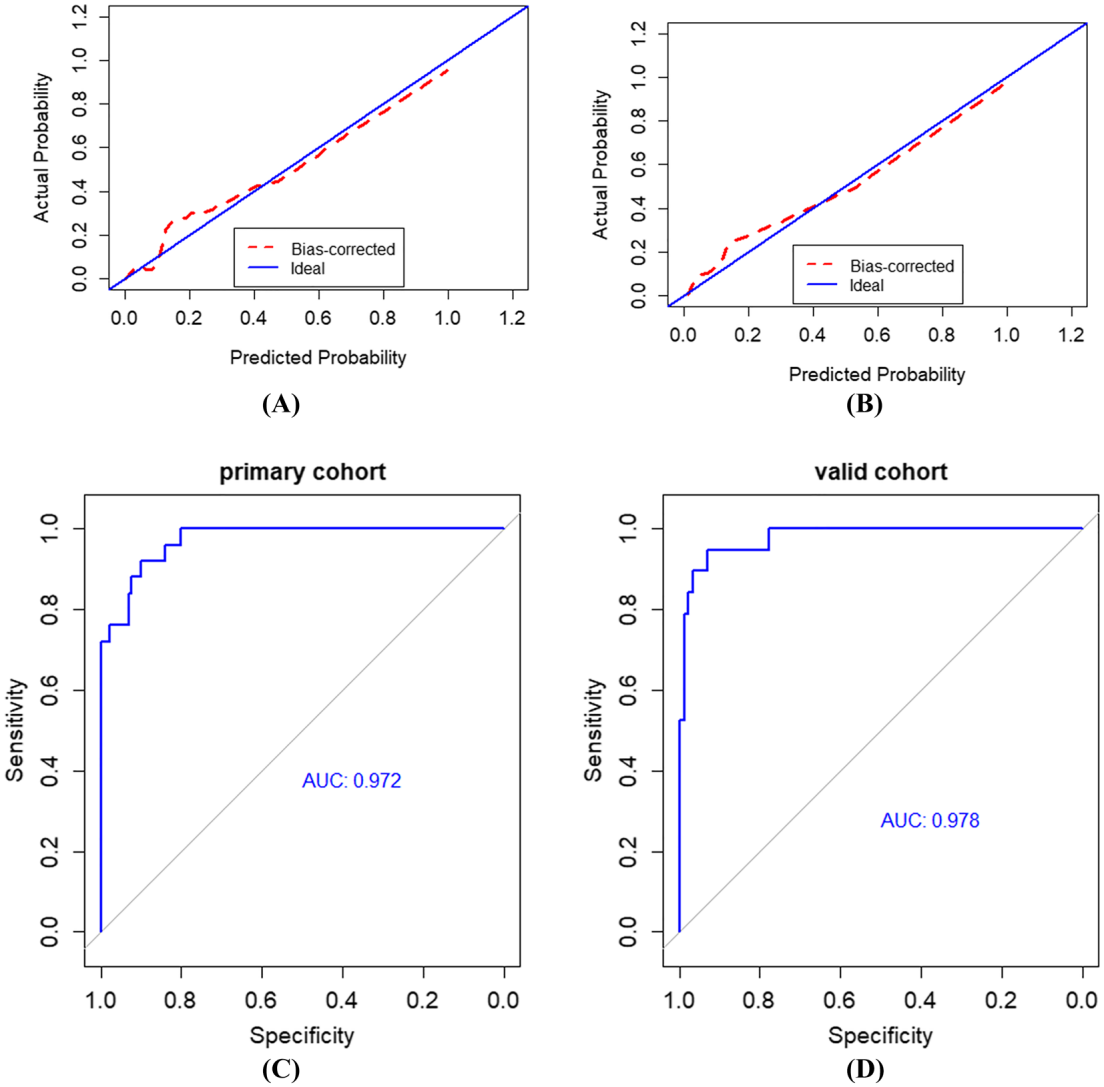
Calibration curves and ROC curves of the radiomics nomogram. **A**, **B** Calibration curves of the radiomics nomogram in the primary and validation cohorts, respectively. **C**, **D** ROC curves of the radiomics nomogram in primary and validation cohorts, respectively

The calibration curve of the radiomics nomogram for the probability of severe and critical condition of COVID-19 patients indicated good agreement between prediction and observation models in the primary cohort (Fig. 5A), which was then confirmed in the validation cohorts (Fig. 5B). The area under the curve of ROC (Fig. 5C, D) for identifying the severe and critical patients based on the radiomic nomogram was 0.972 and 0.978 in the primary and validation cohorts, respectively. In primary cohort, the accuracy, sensitivity and specificity for evaluation of the clinical condition were 0.897, 0.880 and 0.900, respectively. In validation cohort, the corresponding accuracy, sensitivity and specificity were 0.923, 0.894 and 0.929.

### Clinical use

**Fig. 6:**
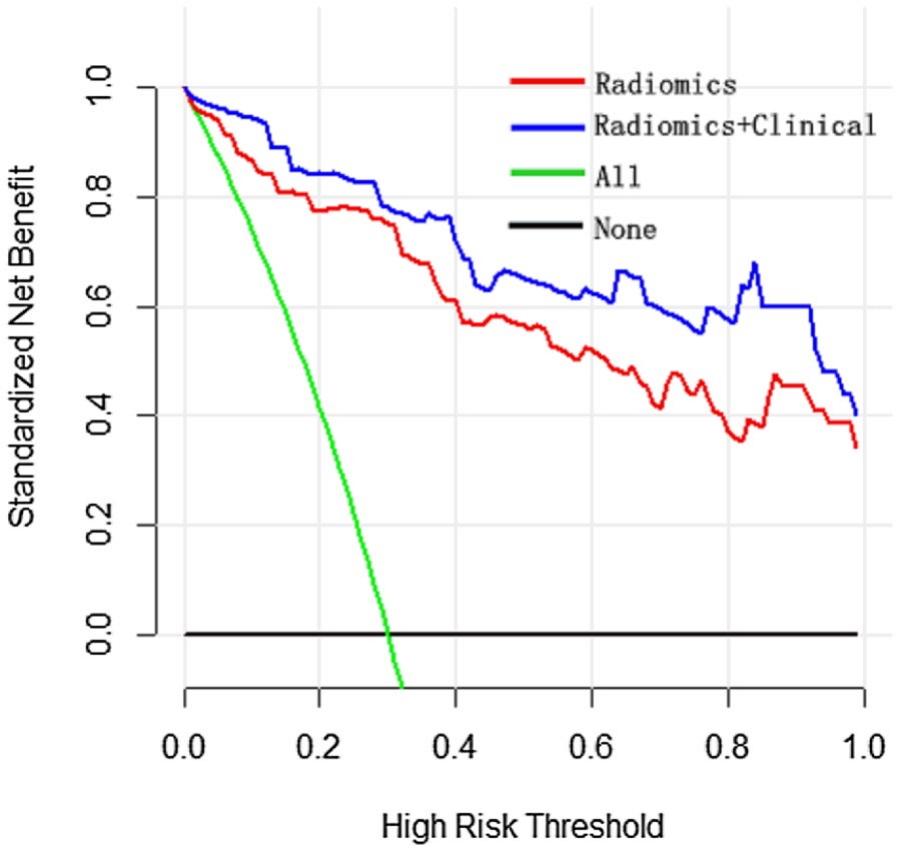
Decision curve analysis for the radiomics nomogram. The *x*-axis represents the threshold probability and the *y*-axis measures the net benefit. The blue line represents the model combines the clinical features with radiomics scores and the pink line represents the radiomics nomogram. The green line assumes that all patients are severe COVID-19. Thin black line hypothesizes that all patients are non-severe COVID-19. The calculation of net benefit was performed by subtracting the proportion of false positive from proportion of true positive in all patients, weighting with the relative harm of giving up treatment compared with the negative consequence of an unnecessary treatment [24]. The calculation of relative harm was performed by (Pt/(1Pt)), Pt is the threshold probability, where the expected benefit of treatment is equal to the expected benefit of avoiding treatment. According to the decision curve, using the method which combines radiomics with clinic information to predict the probability of severe COVID-19 always adds more benefit than other three methods (only including radiomics, or the teat-all-patients scheme, or treat-none scheme). For example, if the personal threshold probability of a patient is 80% (the patient is willing to receive treatment if his probability of severe COVID-19 is > 80%), then the benefit is about 0.6 when using the method that combines radiomics with clinic to decide whether to undergo treatment, which exhibits the best benefit compared with other three methods

The decision curve analysis for the radiomics nomogram is shown in Fig. 6. The decision curve showed that if the threshold probability of a patient is more than 3%, using the radiomics nomogram to identify severe patients adds more benefit than either treat all as severe patients or non-severe patients. When radiomics signature was combined with clinical risk factors (radiomics nomogram), an improved benefit net was achieved.

## Discussion

In this study, we developed and validated a radiomics nomogram based on the quantitation of lung abnormalities on CT images caused by COVID-19 to identify the severe patients for guiding a prompt management and treatment. The radiomics nomogram incorporates three items of the radiomics signature, comorbidity and abnormal WBC counts. The radiomics signature successfully stratified patients according to their clinical conditions (severe or non-severe). The use of multi-task Unet network, which could segment the lesion or lung abnormalities related to COVID-19 automatically, increased the potential value of the radiomics nomogram in evaluating the clinical condition of patients with COVID-19.

Previous studies [18, 19, 24] have demonstrated CT-based radiomics as a superior tool for screening potential new cases of COVID-19, and had a good prediction on discriminating COVID-19 and non-COVID-19 pneumonia or other types of viral pneumonia. Huang et al. [19] summarized 154 patients with viral pneumonia (including 65 cases of influenza pneumonia and 89 cases of COVID-19) to develop a CT-based radiomics model; the results showed radiomics model had a satisfactory performance in distinguishing influenza pneumonia and COVID-19. Chen et al. [24] used COVID-19 risk score combining chest CT radiomics to identify COVID-19 patients from other viral pneumonia. The result showed that the risk score combining radiomics features and clinical variables had an excellent performance in identifying COVID-19 patients with a high AUC value (0.979) and precision value (0.969) in validation set. Nevertheless, it is of great necessity to assess the severity of patients with COVID-19 before treatment, which may greatly determine the clinical prognosis. We first assessed the lung abnormalities associated with COVID-19 by quantitative analysis, and then developed and validated a radiomics signature to identify severe COVID-19 patients. The results in the present study uncovered that the radiomics signature could get a better performance in discriminating the severity of COVID-19 patients with an AUC of 0.943 in primary cohort, which was then further confirmed in validation cohort with an AUC of 0.941. Thus, the radiomics signature was effective for the identification of non-severe and severe type COVID-19 patients. Notably, when combined with clinical risk factors including comorbidity and abnormal WBC counts, the discrimination potency was improved with an AUC of 0.972 and 0.978 in the primary and validation cohorts, respectively. Thus, we think that the noninvasive radiomics signature, which makes most of the chest CT images, may serve as a practical method for the identification of non-severe and severe type COVID-19 patients.

The radiomics signature includes four parameters of pleural thickening, total volume of the lesion, ratio of consolidation volume to whole lung volume and ratio of lesion volume to whole lung volume, which were obtained automatically by computer-aided system or multi-task Unet network. Presently, COVID-19 has reached the stage of a pandemic, which contributed to an extreme shortage of clinicians and radiologists. The application of artificial intelligence technology or computer-aided system, a noninvasive, fast, reproducible technique, to assess the COVID-19 could alleviate the insufficiency of radiologists to some extent. Furthermore, patients with COVID-19 would benefit from a timely and accurate assessment of the severity through radiomics signature before getting a prompt and proper treatment.

It is unexpected that increased total volume of the lesion, ratio of consolidation volume to whole lung volume and ratio of lesion volume to whole lung volume are associated with severe COVID-19 patients. The more extensive involvement of lung parenchymal, the more severe condition it would be. The appearance of GGO indicates that alveolar cavity is partially filled by fluid and cells to the layer against the alveolar walls [25], while the consolidation sign demonstrates that the disease progresses due to further accumulation of exudates in alveolar cavity and aggravation of interstitial edema [25]. The chest CT features of COVID-19 are manifested as multiple patchy GGOs with or without consolidation distributed in subpleural areas of bilateral lungs [8]. When the volume of consolidation increases, more alveolar cavities are filled completely with exudates, resulting in dysfunction of oxygen exchange and oxygenation. Then a respiratory failure occurs, which is presented as a severe condition. Above all, our study quantified the lesion of GGO and consolidation to investigate its value in the identification of severe patients with COVID-19, and to build a useful radiomics signature for clinicians.

Additionally, clinical features including comorbidity and abnormal WBC counts were independent risk factors contributing to worse clinical condition of patients with COVID-19. According to a previous study, the presence of comorbidity is an essential factor in determining the prognosis of several diseases, especially pneumonia [26]. Therefore, we also have taken comorbidity into consideration in the present study and found a positive correlation with the severity of COVID-19, which was consistent with the previously study [27]. CRP is an important inflammatory index. Although a significant difference in CRP increase was indicated by univariate analysis in primary and validation cohorts, it was not an independent predictor for the identification of clinical condition of COVID-19 in this study. The main reasons may be that (1) CRP is a common signal for responding to inflammation; (2) the change of CRP is analyzed as a categorical variable, which may lead to a subtle bias difference. Moreover, viral infections in the human body primarily involve damage to the immune system, which presents as decrease in the absolute number of lymphocytes and leukocyte [28]. In this study, we found that leukocyte and lymphocytes differed between severe and none severe patients with COVID-19, which is consistent with the study of Wang et al. [27]. In addition, WBC (leukocyte) is an independent predictor for the identification of clinical condition of COVID-19. Interestingly, 9 severe patients presented with an increased WBC counts, which may be ascribed to other infections, such as bacterium. Comprehensively, a severe and critical patient with COVID-19 may be caused by cytokine storm, comorbidity with various infections 9 patients with increased WBC counts and immune dysfunction. In a word, incorporating clinical features into radiomics nomogram could improve its diagnostic value of non-severe and severe cases with COVID-19.

The most important application of the radiomics nomogram is to guide management and treatment of patients with COVID-19, especially for severe type cases who need additional treatment or care. According to recent reports and recommendations, severe patients with COVID-19 need hospitalized therapy. Besides antiviral therapy, some additional treatment should be added for severe patients [27, 29]. To block cytokine storm, a blood-purifying therapy including plasmapheresis and hemoperfusion is recommended, which can reduce the damage of inflammatory reaction to the body or lung [30]. If possible, convalescent plasma therapy could be a preferred scheme for the treatment of severe patients [30]. Using the nomogram, we can quickly and precisely identify the non-severe and severe patients with COVID-19, and prompt a timely additional treatment and care to improve prognosis. On the other hand, COVID-19 is a dynamic disease [31]; a quantitative radiomics nomogram is helpful to follow up the changes of patients after treatment. To justify the clinical practicability of radiomics nomogram, decision curve analysis was applied in this study. This novel method offers an insight into clinical consequences based on threshold probability, from which the net benefit could be derived (net benefit is defined as the proportion of true positives minus the proportion of false positives, weighted by the relative harm of false-positive and false-negative results). The decision curve in our study showed that if the threshold probability of a patient was more than 3%, using the radiomics nomogram to identify non-severe or severe patients added more benefit than either treat all as severe patients or non-severe patients.

Admittedly, our study has several limitations. The sample size in our cohort is relatively small. The relationship of radiomics to prognosis has not been studied due to time limitation. Thus, a further study with more cases and prolonged period should be conducted in the future.

## Conclusion

In conclusion, we present an easy-to-use radiomics nomogram to identify the severe patients of COVID-19 for guiding a prompt management and treatment. We believe that both clinicians and COVID-19 patients could greatly profit from the use of the radiomics nomogram.

## Data Availability

All data generated or analyzed during this study are included in this published article.

## Abbreviations

COVID-19: Coronavirus disease 2019
CRP: C-reactive protein
CT: Computed tomography
dFOV: Display field of view
GGO: Ground-glass opacity
HU: Hounsfield unit
LASSO: Least absolute shrinkage and selection operator
RT-PCR: Real-time reverse transcriptase polymerase chain reaction
WBC: White blood cell
